# Confirmation of intestinal and bladder perforations in a peritoneal dialysis patient using SPECT/CT: a case report and review of literature

**DOI:** 10.3389/fmed.2023.1327295

**Published:** 2024-01-08

**Authors:** Xinchao Zhang, Yujing Hu, Fenglian Jing, Congna Tian, Qiang Wei, Kang Li, Lu Zheng, Jiale Liu, Jingjie Zhang, Yanzhu Bian

**Affiliations:** ^1^Department of Nuclear Medicine, Hebei General Hospital, Shijiazhuang, China; ^2^Department of Nuclear Medicine, The Fourth Hospital of Hebei Medical University, Shijiazhuang, China

**Keywords:** peritoneal dialysis, intestinal perforation, bladder perforation, SPECT/CT, ^99m^Tc

## Abstract

**Background:**

Peritoneal dialysis (PD) is a common treatment method for patients with renal failure. While peritonitis and tube floating migration are commonly observed complications, visceral perforation caused by PD is relatively rare. We present a case report of a patient undergoing PD due to renal failure, who encountered two instances of visceral perforation. In both occurrences, Single-Photon Emission Computed Tomography/Computed Tomography (SPECT/CT) played a pivotal role in providing accurate diagnoses and precise localization of the perforation sites. This report underscores the paramount significance of SPECT/CT in diagnosing visceral perforations in the context of PD.

**Case presentation:**

A 73-year-old elderly male has been undergoing PD for 1 year due to renal failure. Recently, there has been impaired drainage of the PD catheter. The clinical team suspected the occurrence of peritonitis. The patient underwent a ^99m^Tc Sodium Pertechnetate (^99m^Tc-NaTcO_4_) SPECT/CT examination, which identified intestinal perforation. After 20 days of conservative treatment, a SPECT/CT follow-up examination revealed the resolution of the intestinal perforation, but a new bladder perforation emerged. The dialysis catheter was methodically and gradually withdrawn in stages while simultaneously performing bladder decompression. Following these interventions, the patient remained free from peritonitis and cystitis.

**Conclusion:**

The utilization of SPECT/CT proved to be highly valuable in the accurate diagnosis of visceral perforation, a relatively rare complication observed in PD patients.

## Introduction

Peritoneal dialysis (PD) is widely utilized in patients with renal failure due to its high level of safety and efficacy. Pain and catheter leakage are the most commonly reported issues associated with PD (1). However, the occurrence of catheter penetration through the intestinal wall, leading to intestinal perforation, is a rare complication that can have severe consequences, including peritonitis, impaired drainage, and severe diarrhea (2). Diagnosing perforation-related peritonitis caused by PD is often challenging, particularly when it comes to identifying and locating the specific site of perforation. Conventional imaging studies often have limitations in detecting visceral perforation caused by PD, while the use of contrast agents for imaging procedures may potentially increase the renal burden in patients (3). Here, we report the case of intestinal and bladder perforation that occurred after PD. The utilization of Single-Photon Emission Computed Tomography/Computed Tomography (SPECT/CT) imaging played a pivotal role in accurately diagnosing these visceral perforations, thereby highlighting its diagnostic significance in such cases.

## Case presentation

A 73-year-old man underwent maintenance PD for 1 year due to renal failure. Radical resection of rectal cancer with terminal ileostomy was performed 8 months ago due to rectal cancer, and the ileostomy was closed 2 months ago. The PD tube was found to be dysfunctional 5 days ago and failed to drain 1 day ago. Laboratory results showed hypokalemia (serum potassium: 2.9 mmol/L), hypocalcemia (serum calcium: 2.03 mmol/L), elevated serum creatinine (636.8 μmol/L), elevated blood urea (10.6 mmol/L), and a glomerular filtration rate of 6.83 mL/min. Calcitonin level was measured at 3.547 ng/mL, and C-reactive protein was elevated at 151.23 mg/L. The clinical team suspected peritonitis concurrent with hypokalemia and hypocalcemia. The patient received treatment with cefuroxime for antimicrobial therapy, along with blood dialysis for endotoxin clearance. Subsequent retesting showed normalization of calcitonin and C-reactive protein levels.

**Figure 1:**
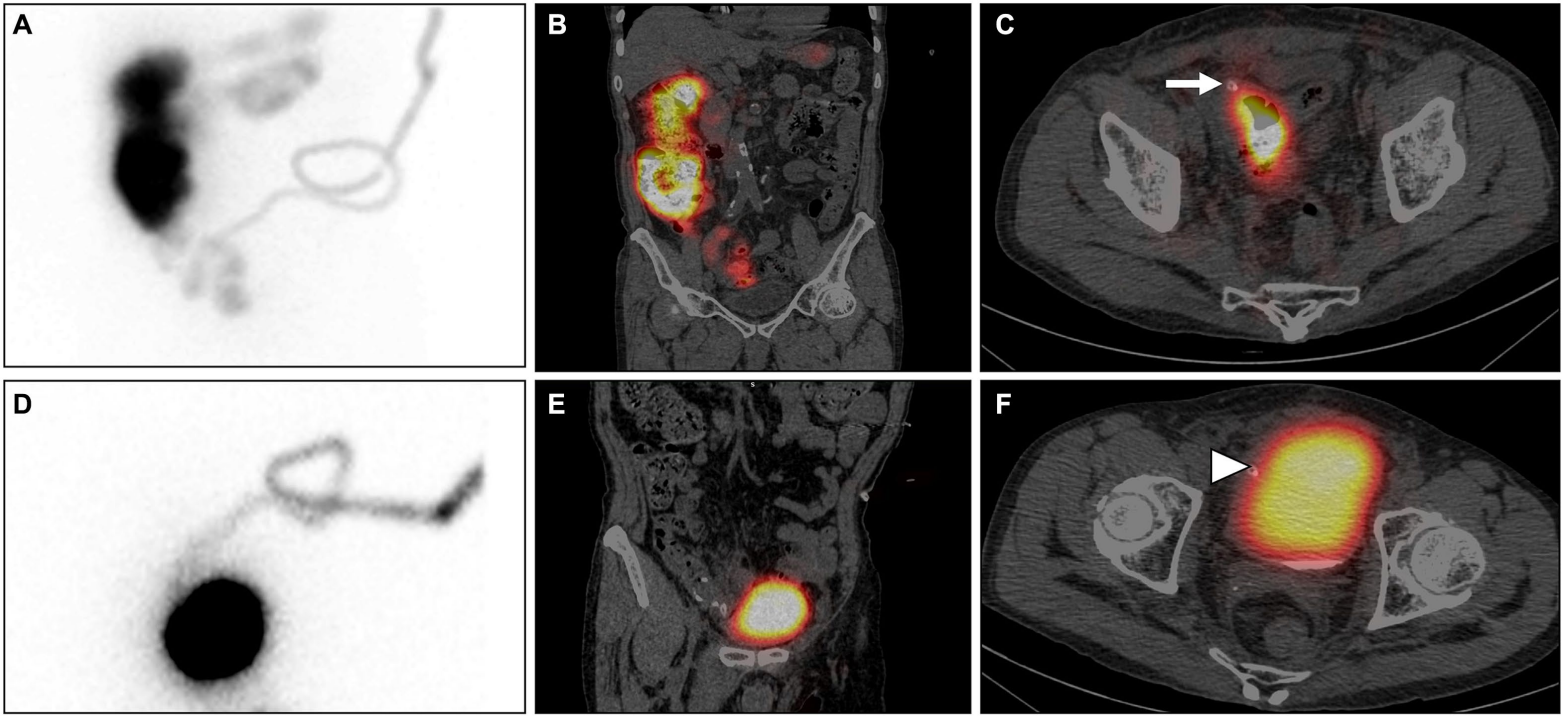
99mTc-NaTcO4 SPECT/CT: The Maximum Intensity Projection (MIP) image **(A)** and coronal SPECT/CT **(B)** demonstrate radiotracer uptake in the intestines, while the axial SPECT/CT **(C)** indicates the site of perforation at the terminal ileum (arrow). After conservative treatment, re-examination with SPECT/CT shows radiotracer uptake in the bladder and no radiotracer uptake in the intestines on the MIP **(D)** and coronal SPECT/CT **(E)**. The axial SPECT/CT **(F)** confirms the presence of bladder perforation (arrowhead).

SPECT/CT imaging was conducted by nuclear medicine physicians, precisely 30 min after the infusion of 3 mCi of ^99m^Tc Sodium Pertechnetate (^99m^Tc-NaTcO_4_) into the PD catheter. The Maximum Intensity Projection (MIP) image (Figure 1A) and coronal SPECT/CT (Figure 1B) demonstrate radiotracer uptake in the intestines, while the axial SPECT/CT (Figure 1C) indicates the site of perforation at the terminal ileum (arrow). The imaging findings confirmed the occurrence of intestinal perforation in the patient. After an extensive multidisciplinary consultation, a decision was reached to temporarily refrain from catheter removal. The patient was commenced on a course of conservative treatment, concurrently bolstered by intensified nutritional support.

Following a conservative treatment duration of 20 days, a subsequent SPECT/CT follow-up examination was performed. Intriguingly, no presence of imaging agent was detected within the intestinal lumen; instead, it was observed to traverse through the dialysis tube and accumulate within the bladder (Figure 1D, MIP; Figure 1E, coronal SPECT/CT; Figure 1F, axial SPECT/CT). The imaging findings indicated spontaneous resolution of the bowel perforation, accompanied by the emergence of a new bladder perforation (arrowhead). Following another comprehensive hospital-wide consultation, considering the preliminary formation of a sinus tract at the site of intestinal perforation, a decision was reached to plan the gradual removal of the PD catheter at a suitable time. Concurrently, urinary catheterization was employed to alleviate bladder pressure and promote spontaneous closure of the bladder perforation. Subsequent urine analysis revealed hematuria (3+), glycosuria (1+), proteinuria (3+), leukocyturia (2+), and a notable count of red blood cells (48,181/μL) and white blood cells (19,673/μL). The antimicrobial regimen was adjusted to piperacillin/tazobactam for continued infection management. Alongside blood purification therapy, the PD catheter was incrementally withdrawn by 2 to 3 centimeters every 3 days, with a complete removal achieved after a duration of 1.5 months. The patient experienced a favorable recovery without any signs of cystitis or peritonitis.

## Discussion

Malfunctions resulting from the displacement or distortion of the PD catheter tip, as well as catheter clot formation, have been identified in the literature as the primary causes of inadequate drainage (4, 5). PD-related perforations of adjacent organs are rare. A study conducted by Yang JY revealed that the incidence of intestinal perforation in the United States between 1992 and 2005 was approximately 0.4%. The 30-day mortality rates following intestinal perforation were reported to be 42.3% for all cases of intestinal perforation (6). In our analysis of cases over the past decade involving PD complicated by visceral perforation, we identified 18 reported instances in the literature. Among these cases, intestinal perforation was the most common, accounting for 13 cases, while bladder perforation occurred in only one case. Other types of perforations included venous, gallbladder, and heart perforations.

While visceral perforation caused by PD is exceedingly rare, once catheter-related complications occur, they may lead to permanent technical malfunction of the drainage catheter, ultimately necessitating permanent conversion to hemodialysis in up to 20% of patients (7). PD procedures can be classified based on different catheter implantation techniques, including direct visualization insertion and precise percutaneous puncture insertion. Direct visualization insertion involves the real-time observation and insertion of the PD catheter during surgery, typically encompassing open surgical or laparoscopic-assisted approaches. Percutaneous puncture insertion, falling under the category of closed PD, entails the insertion of the PD catheter through skin puncture and catheter-guided techniques, often utilizing methods such as urethral catheter insertion or surgical catheter placement. Early complications refer to those arising during the perioperative period. Among the most concerning early complications, visceral injuries have been reported in both open and closed insertion techniques, with symptomatology sometimes being misleading (8). The probability of visceral perforation varies among different catheter placement techniques, and four retrospective studies have demonstrated that despite differences in placement methods, the incidence of visceral perforation remains below 1% (9–12). Furthermore, the research findings suggest that visceral perforation is more common when using blind percutaneous insertion for PD catheters. Consequently, the use of techniques allowing direct visualization (such as laparoscopy or open insertion surgery) is recommended for patients with a history of prior abdominal surgery, severe or recurrent peritonitis, morbid obesity, or anatomical anomalies (13).

In clinical follow-up studies, early symptoms of intestinal perforation include mild abdominal discomfort or fever, accompanied by drainage of yellow fluid from the dialysis catheter (14); watery diarrhea and recurrent peritonitis (15); acute abdomen, hypotension, and severe vomiting (16). There have been limited reports of bladder perforation caused by PD, hence its clinical manifestations remain inconclusive. The literature indicates that visceral injuries resulting from PD catheter insertion exhibit varying symptoms, although a significant portion of these symptoms may remain inconspicuous (17). Therefore after the placement of the peritoneal dialysis catheter, its intra-abdominal position and buoyancy should be examined through imaging methods to ensure the appropriateness of the catheter placement. In cases where there is suspicion of perforation or peritonitis, it is advisable to retain an appropriate amount of peritoneal dialysis fluid to prevent exacerbation of perforation or inflammation. In addition for patients with a history of previous major abdominal surgeries or episodes of peritonitis, the placement of PD catheters is not recommended. This is due to the potential augmentation of organ adhesion during the insertion process, consequently leading to an increased likelihood of catheter obstruction.

There have been few reports on the diagnosis of PD with adjacent tissue perforation using SPECT/CT, only subcutaneous leakage and pleuroperitoneal fistula, and no case report of organ perforation (18–20). The commonly employed diagnostic method for visceral perforation is X-ray contrast imaging. However, the administration of contrast agents undoubtedly imposes an additional burden on the patient’s kidneys and is not conducive to performing consecutive dynamic scans. In the early stages of intestinal perforation, before the formation of subdiaphragmatic free gas, injecting a radioactive tracer into the peritoneal dialysis catheter enables SPECT to reveal whether the imaging agent has entered the intestine, facilitating early diagnosis of intestinal perforation. Its sensitivity and specificity are higher than CT, albeit with the drawback of requiring contrast agent injection, making the procedure slightly more complex. Gastrointestinal perforation can be diagnosed through CT, but a definitive diagnosis typically requires a certain duration of perforation or the presence of free intraperitoneal gas before becoming evident. Peritoneal dialysis patients exhibit increased intestinal wall edema, permeability, and fragility, making them more prone to intestinal wall perforation and injury compared to the normal intestine. Additionally, they are more susceptible to peritonitis. Hence, early diagnosis of perforation is crucial. The imaging agent utilized in SPECT can enter the gastrointestinal tract through the peritoneal dialysis catheter in the early stages of intestinal perforation. Therefore, it enables the early diagnosis of perforation, allowing for earlier detection and localization compared to conventional CT. This is precisely the advantage of SPECT reported in this study. In addition the chemical quantity of the imaging agent used in SPECT imaging is extremely minimal, to the extent that it can be disregarded, and it will not impose an additional burden on the kidneys of peritoneal dialysis patients.

The radiopharmaceutical dose utilized in ^99m^Tc-NaTcO_4_ SPECT/CT imaging is characterized by its low radiation dosage, thereby mitigating any exacerbation of renal strain in patients. Additionally, it facilitates uninterrupted dynamic scanning, enabling precise localization of the perforation site. This technique plays a paramount role in the diagnosis and precise localization of visceral perforations, underscoring its significant clinical significance. As previously mentioned, the mortality risk for patients with visceral perforation is significantly heightened; thus, early detection of such perforations becomes imperative in order to mitigate mortality rates. Undoubtedly, SPECT/CT demonstrates notable diagnostic efficacy in the detection of PD complicated by visceral perforation.

## Conclusion

Visceral perforations caused by PD are exceedingly rare, with the majority involving intestinal perforations. Patients often present with atypical clinical manifestations. For patients with a history of prior abdominal surgeries or peritonitis, it is recommended to undergo visually guided PD techniques or consider transitioning to hemodialysis instead of PD. SPECT/CT plays a crucial role in the diagnosis and localization of visceral perforations, given the elevated mortality rate associated with such occurrences.

## Data availability statement

The raw data supporting the conclusions of this article will be made available by the authors, without undue reservation.

## Ethics statement

The studies involving humans were approved by the Institutional Review Board of Hebei General Hospital. The studies were conducted in accordance with the local legislation and institutional requirements. The participants provided their written informed consent to participate in this study. Written informed consent was obtained from the individual(s) for the publication of any potentially identifiable images or data included in this article. Written informed consent was obtained from the participant/patient(s) for the publication of this case report.

## Author contributions

XZ: Writing – original draft, Writing – review & editing. FJ: Writing – review & editing. YH: Writing – review & editing. CT: Writing – review & editing. QW: Writing – review & editing. KL: Writing – review & editing. LZ: Data curation, Writing – review & editing. JZ: Writing – review & editing. YB: Visualization, Writing – original draft, Writing – review & editing. JL: Data curation, Writing – original draft.
